# Identification of Key Genes of Human Advanced Diabetic Nephropathy Independent of Proteinuria by Transcriptome Analysis

**DOI:** 10.1155/2020/7283581

**Published:** 2020-06-26

**Authors:** Fanghao Cai, Xujie Zhou, Yan Jia, Weijian Yao, Jicheng Lv, Gang Liu, Li Yang

**Affiliations:** ^1^Renal Division, Peking University First Hospital, China; ^2^Peking University Institute of Nephrology, Key Laboratory of Renal Disease, Ministry of Health of, China; ^3^Key Laboratory of Chronic Kidney Disease Prevention and Treatment (Peking University), Ministry of Education, Beijing 100034, China

## Abstract

**Background:**

Diabetic nephropathy (DN) is the leading cause of ESRD. Emerging evidence indicated that proteinuria may not be the determinant of renal survival in DN. The aim of the current study was to provide molecular signatures apart from proteinuria in DN by an integrative bioinformatics approach.

**Method:**

Affymetrix microarray datasets from microdissected glomerular and tubulointerstitial compartments of DN, healthy controls, and proteinuric disease controls including minimal change disease and membranous nephropathy were extracted from open-access database. Differentially expressed genes (DEGs) in DN versus both healthy and proteinuric controls were identified by limma package, and further defined by Gene Ontology (GO) and Kyoto Encyclopedia of Genes and Genomes (KEGG) pathway analysis. Hub genes were checked by protein-protein interaction networks.

**Results:**

A total of 566 glomerular and 581 tubulointerstitial DEGs were identified in DN, which were commonly differentially expressed compared to normal controls and proteinuric disease controls. The upregulated DEGs in both compartments were significantly enriched in GO biological process associated with fibrosis, inflammation, and platelet dysfunction, and largely located in extracellular space, including matrix and extracellular vesicles. Pathway analysis highlighted immune system regulation. Hub genes of the upregulated DEGs negatively correlated with estimated glomerular filtration rate (eGFR). While the downregulated DEGs and their hub genes in tubulointerstitium were enriched in pathways associated with lipid metabolism and oxidation, which positively correlated with eGFR.

**Conclusions:**

Our study identified pathways including fibrosis, inflammation, lipid metabolism, and oxidative stress contributing to the progression of DN independent of proteinuria. These genes may serve as biomarkers and therapeutic targets.

## 1. Introduction

Diabetic nephropathy (DN) is the leading cause of chronic kidney disease (CKD) and end stage renal disease (ESRD) [1, 2]. Although urinary protein is recognized as the major culprit to the progression of DN [3], it was observed that renal outcomes were worse in patients with DN than those with nondiabetic renal diseases irrespective of proteinuria [4, 5]. Recent studies reported renal insufficiency could happen before the presence of albuminuria, which also indicated that the dominant feature of DN could be progressive renal decline rather than proteinuria [6, 7]. Thus, identifying molecules not relating to proteinuria may shed additional light on DN pathogenesis. However, integrative analysis investigating such molecules has not been reported.

Renal biopsy captures pathological characteristics of the disease and the gene expression profiles of renal tissue provide unbiased comprehensive understanding of the molecular mechanisms. Several transcriptome analyses have been taken in DN and they observed molecules and pathways aberrantly regulated in DN such as matrix expansion, vascular damage, and inflammation [8–10]. Results were somehow inconsistent. And these studies focused on renal glomerular or tubulointerstitial lesions differently. However, both glomeruli and tubules are seriously affected in DN, and it is unclear how they relatedly or differently contribute to DN development. In addition, the controls were normal renal tissues, whether the pathways were the initiating mechanism or secondary injury to proteinuria remains to be explored.

In the current study, to further rule out the confounding role of proteinuria, we included datasets from both DN and proteinuric controls including minimal change disease (MCD) and membranous nephropathy (MN) [11, 12]. And we separately evaluate glomerular and tubulointerstitial compartments. The study aims to provide molecular signatures apart from proteinuria in DN by the integrative bioinformatics approach.

## 2. Materials and Methods

### 2.1. Microarray Data

Microdissection transcriptome data were obtained from the public genomics data repository GEO (https://www.ncbi.nlm.nih.gov/geo/). “Diabetic nephropathy OR DN”, “Membranous nephropathy OR membranous glomerulonephritis OR MGN OR MN”, and “minimal change diseases OR MCD” were searched, respectively, and kidney biopsy samples were from the European Renal cDNA bank (ERCB) cohort, which was established to collect kidney biopsy tissue for gene expression analysis, were studied. After excluding duplicated submissions, GEL files from GSE47183 [13] and GSE37463 [14] based on both platform GLP11670 (Affymetrix Human Genome U133 Plus 2.0 Array) and GLP14663 (Affymetrix Human Genome U133A Array), and GSE104954 [15] based on both platform GLP22945 and GLP24120 were analyzed. Altogether, 77 glomerular microdissection samples including 14 with diabetic DN, 15 with MCD, 21 with MN, and 27 from living donors as healthy controls and 69 tubulointerstitial microdissection samples including 17 with DN, 13 with MCD, 18 with MN, and 21 from healthy controls were enrolled. As we aimed to focus on typical DN, we therefore did not perform our search by “DKD” (diabetic kidney disease), since it may contain various kidney injuries associated directly or indirectly with diabetes.

According to the published study which originally submitted the microdissected glomeruli microarray, the urinary protein level of DN, MN, and MCD cases were 3.1 ± 2.7 g/d, 4.6 ± 3.2 g/d, and 6.7 ± 5.8 g/d, respectively [13]. The microdissected renal tubule microarray data were obtained from MN and MCD patients with nephrotic syndrome [15]. Therefore, the microarray information that we used in the current study was from DN, MN, and MCD patients of comparable amount of proteinuria. However, DN patients seemed to have worse renal function, as DN in GSE47183 had a lower eGFR value of 44 ± 25 ml/min per 1.73m^2^, compared to MN and MCD patients (eGFR 89 ± 41 and 101 ± 34 ml/min per 1.73m^2^) [13].

### 2.2. Microarray Datasets Preprocessing and Differentially Expressed Gene (DEG) Identification

Affymetrix CEL files were normalized using the robust multiarray average method, log2 transformed using R software (version 3.6.1). Expression data with shared probes by the Human Genome U133 Plus 2.0 Array and Human Genome U133A Array were combined and batch corrected by “removebatcheffects” function in Limma package [16]. Probes were annotated at Entrez Gene level. And those without gene symbols were removed or genes with more than one probes were averaged. Limma package was used to screen DEGs in the glomerular and tubulointerstitial compartments between healthy controls, patients with MN and MCD versus DN. DEGs were defined as adjusted *P* < 0.05 and fold changes >1.5.

### 2.3. Gene Ontology (GO) and Pathway Enrichment Analysis

An online bioinformatics tool DAVID version 6.8 (https://david.ncifcrf.gov/) was used for GO enrichment analysis and Kyoto Encyclopedia of Genes and Genomes (KEGG) pathway analysis. GO analysis enables the annotation of cellular component (CC), biological process (BP), and molecular function (MF). KEGG pathway illustrates the path of the gene cluster and associated functions. GO annotations relating to other diseases or organs were excluded. Gene count >2 and *P* < 0.05 were set as the threshold.

### 2.4. Protein-Protein Interaction (PPI) Analysis

The Search Tool for the Retrieval of Interacting Genes (STRING) version 11 (http://string-db.org/) was used to construct the PPI networks of DEGs. Interaction score >0.7 was set as the cut-off point. Cytoscape software version 3.6.1 was applied to visualize the PPI network and analyze the interactive relationships. The plugin cytoHubba [17] was used to explore hub genes and subnetworks by topological analysis strategy. The top 30 nodes calculated by the maximal clique centrality (MCC) algorithm were shown as hub genes in the network. The plugin Molecular Complex Detection (MCODE) was performed to identify key clusters.

### 2.5. Correlation Analysis with Renal Function

The correlation between hub gene expression and estimated glomerular filtration rate (eGFR) in DN patients was performed in Nephroseq v5 online platform (http://v5.nephroseq.org/) and analyzed using the spearman correlation coefficient. *P* values <0.05 were considered statistically significant.

## 3. Results

### 3.1. Identification of DEGs Specific in DN

**Figure 1:**
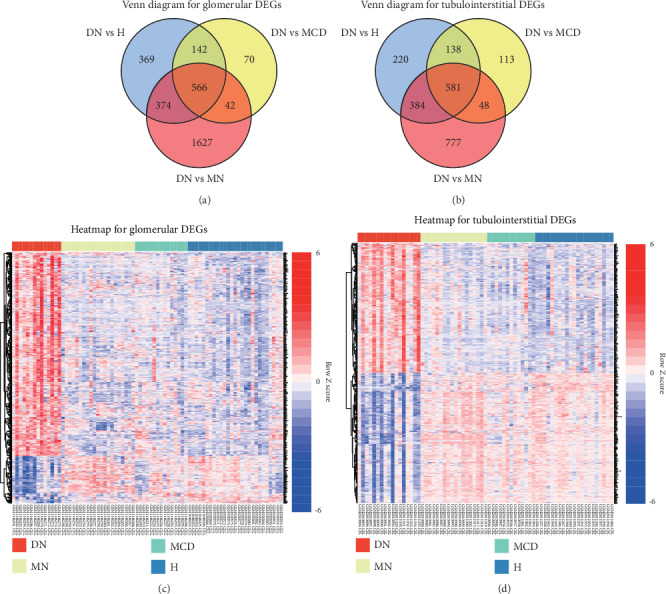
Identification of DEGs in DN compared with healthy controls, proteinuric controls including MCD and MN. Venn diagrams showing the shared glomerular (a) and tubulointerstitial (b) DEGs. Heatmaps of DEGs in glomerular (c) and tubulointerstitial (d) compartments. Red areas represent highly expressed genes, and blue areas represent lowly expressed genes of DN patients compared with those of healthy and disease controls. DEGs: differentially expressed genes; DN: diabetic nephropathy; MN: membranous nephropathy; MCD: minimal change disease; H: healthy controls; vs: versus.

A total of 566 glomerular and 581 tubulointerstitial genes were significantly differentially expressed in DN compared with all of those in healthy controls, MCD and MN (Figures 1(a) and 1(b)). As shown in heatmaps, 453 DEGs were upregulated and 105 DEGs were downregulated in glomeruli (Figure 1(c)). While in tubulointerstitium, there were 287 upregulated and 290 downregulated DEGs in DN compared with proteinuric controls (Figure 1(d)). The complete lists of shared DEGs and the remaining DEGs were presented in Supplementary File 1.

### 3.2. GO Enrichment Analysis of Shared DEGs

**Figure 2:**
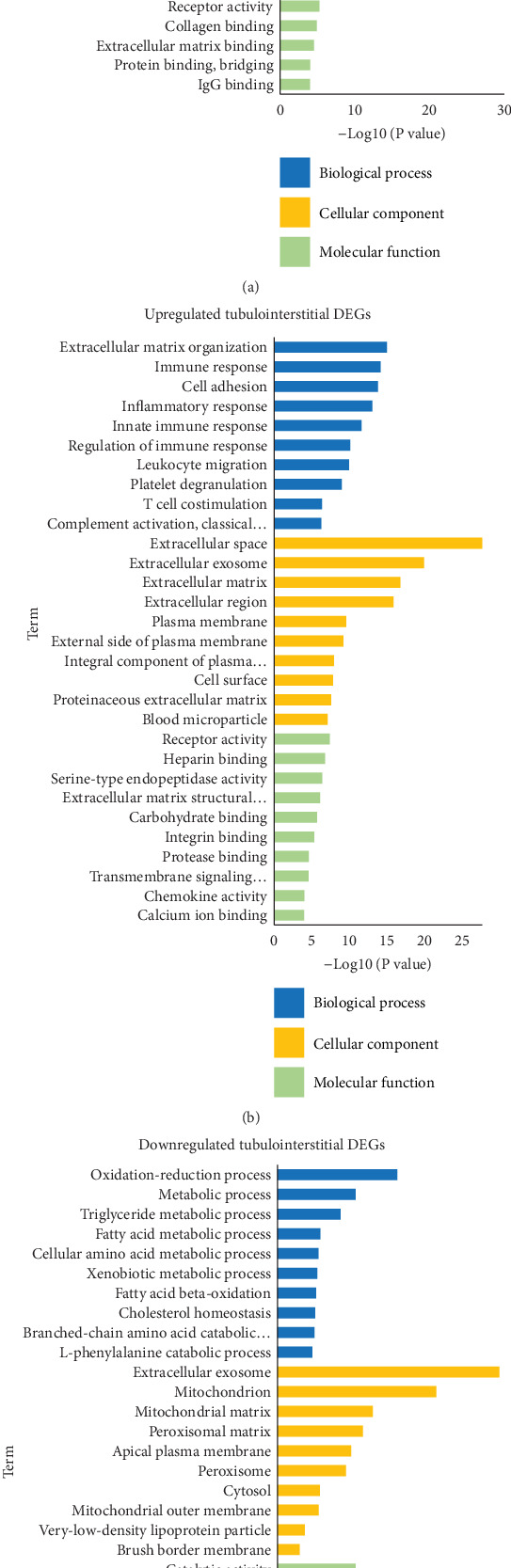
The Top 10 BP, CC, and MF terms of shared DEGs. GO enrichment analysis of upregulated DEGs in glomerular (a) and tubulointerstitial (b) compartments. **(**c**)** GO enrichment analysis of downregulated DEGs in tubulointerstitium. DEGs: differentially expressed genes; BP: biological process; CC: cellular component; MF: molecular function; GO: Gene Ontology.

A total of 113 GO terms in glomeruli and 83 GO terms in tubulointerstitium of upregulated DEGs were identified according to Benjamin adjusted *P* values of <0.05 (Supplementary File 2). As shown in Figure 2(a) and 2(b), in both glomerular and tubulointerstitial compartments, the upregulated DEGs were significantly enriched in BPs associated with fibrosis, inflammation, and platelet dysfunction, including extracellular matrix organization, collagen catabolic process, inflammatory response, immune response, and platelet degranulation and activation. Their CCs were most significantly enriched in extracellular space, including matrix and vesicles, i.e., exosomes and blood microparticles. Moreover, 53 DEGs in both glomeruli and tubulointerstitium located in exosomes, and 9 in microparticles. We further performed enrichment analysis for them. DEGs in exosomes were mainly associated with leukocyte migration, extracellular matrix organization, and platelet degranulation, and DEGs in blood microparticles mainly associated with complement activation, innate immune response, and platelet degranulation (Supplementary File 3). The MFs of upregulated DEGs were involved in extracellular matrix structural constituent, receptor activity and chemokine activity.

For downregulated DEGs, 46 GO terms in tubulointerstitium were identified by the adjusted *P* value (Supplementary File 2). The downregulated DEGs of tubulointerstitium were enriched in the BPs of oxidative-reduction and metabolic process, and these DEGs were localized in cytoplasm, particularly in mitochondria. Their MFs involved catalytic activity and oxidoreductase activity (Figure 2(c)). While the downregulated DEGs of glomeruli were not markedly enriched in any BPs.

### 3.3. Pathway Enrichment Analysis

**Figure 3:**
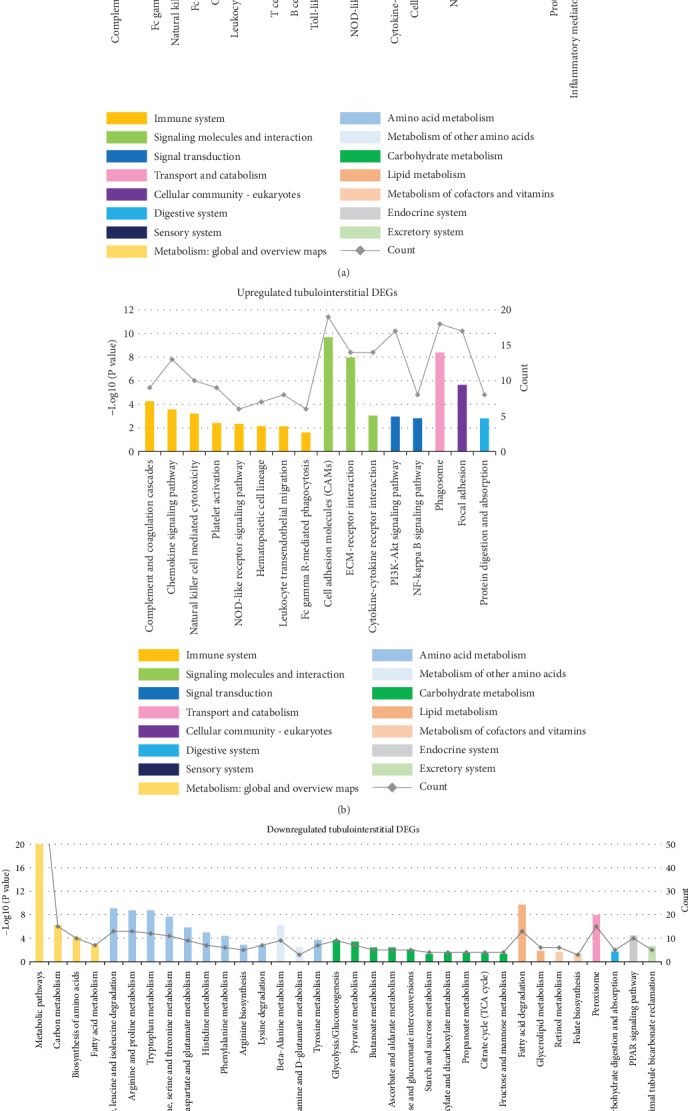
KEGG pathway enrichment analysis. KEGG pathway enrichment analysis of upregulated DEGs in glomerular (a) and tubulointerstitial (b) compartments. **(**c**)** KEGG pathway enrichment analysis of downregulated DEGs in tubulointerstitium. DEGs: differentially expressed genes; KEGG: Kyoto Encyclopedia of Genes and Genomes.

For upregulated DEGs, 13 of 24 (54.2%) enriched pathways in glomeruli and 8 of 16 (50%) in tubulointerstitium were involved in the immune system regulation, including chemokine signaling pathway, complement and coagulation cascades, natural killer cell-mediated cytotoxicity, platelet activation, and NOD-like receptor signaling pathway. Of note, upregulated DEGs in both glomeruli and tubulointerstitium were enriched in NF-kappa B signaling pathway, and PI3K-Akt signaling pathway (Figures 3(a) and 3(b)). The analysis of downregulated DEGs revealed pathways involving carbohydrate metabolism and amino acid metabolism (Figure 3(c)).

### 3.4. PPI Network Analysis and Hub Genes Recognition

**Figure 4:**
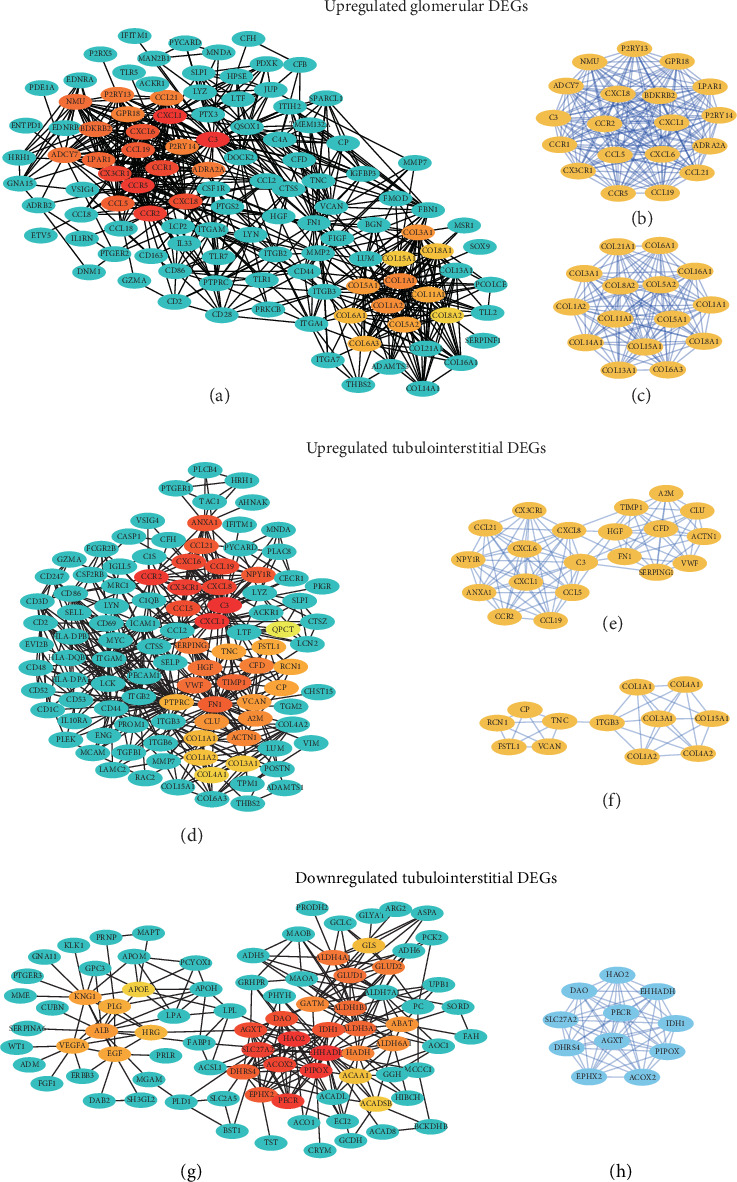
PPI network and modules constructed by cytoscape. PPI network of upregulated DEGs in glomerular (a) and tubulointerstitial (d) compartments and downregulated DEGs in tubulointerstitium (g). Hub genes were selected by MCC algorithms using cytoHubba plugin. The top 30 ranked DEGs were shown with a color scheme from highly essential (red) to essential (yellow). The most (b) and second significant clusters (c) of upregulated DEGs in glomeruli identified by MCODE plugin. The most (e) and second significant clusters (f) of upregulated DEGs in tubulointerstitium. (h) The most significant cluster of downregulated DEGs in tubulointerstitium. DEGs: differentially expressed genes; PPI: protein-protein interaction.

In both glomeruli and tubulointerstitium, the upregulated hub genes identified by the MCC algorithm of interactions were associated with inflammatory (C3, CCR2, CCL5, CXCL1, CCL19, and CCL21) and fibrosis (COL1A1, COL1A2, COL3A1, and COL15A1; Figures 4(a) and 4(d)). Furthermore, MCODE score system identified two clusters, enriched in chemokines and collagens, of the upregulated DEGs (Figures 4(b), 4(c), 4(e), and 4(f)). C3 was in the clusters associated with inflammation and highly interacted with chemokines (Figures 4(b) and 4(e)). The hub genes of decreased DEGs in tubulointerstitium were shown in Figures 4(g) and 4(h), where the most significant cluster consisted of 11 hub genes. We then performed GO analysis for these 11 DEGs, and found that they were located in peroxisomes and mitochondria, and enriched in cellular lipid metabolic process (i.e., EHHADH, HAO2, PECR), fatty acid beta-oxidation (i.e., ACOX2, EHHADH, SLC27A2) and oxidation-reduction process (i.e., DHRS4, DAO, PECR, PIPOX).

**Figure 5:**
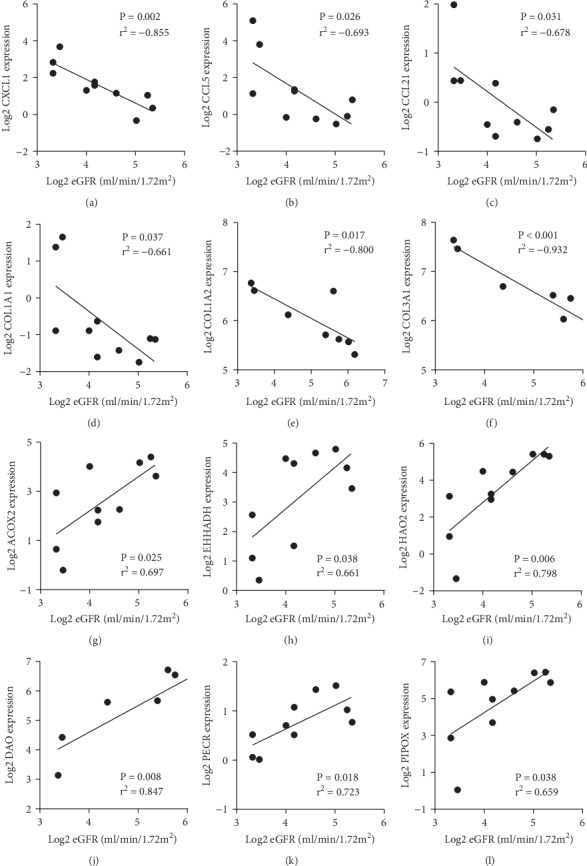
Correlation between eGFR and mRNA expression of hub genes in tubulointerstitium. (a–f) The expression of upregulated genes relating to inflammation or fibrosis negatively correlated with eGFR. (g–l) The expression of downregulated genes regulating lipid metabolism and oxidative processes positively correlated with eGFR. eGFR: estimated glomerular filtration rate.

Further exploring the association of DEGs and kidney function, the mRNA expressions of cytokines (CXCL1, CCL5, and CCL21) and collagens (COL1A1, COL1A2, and COL3A1) were observed to have a negative correlation with eGFR in DN patients (Figures 5(a)–5(f)). While the mRNA expression of genes mediating metabolic (ACOX2, EHHADH, and HAO2) and oxidative-reduction processes (DAO, PECR, and PIPOX) positively correlated with eGFR (Figures 5(g)–5(l)).

## 4. Discussion

Although DN is not considered an inflammatory disease, kidney inflammation could be a key pathophysiological mechanism. In our study, the increased hub genes relating to the inflammation in glomeruli and tubulointerstitium included chemokines, i.e., CCL5, CXCL1, CCL19, and CCL21, chemokine receptor CCR2, and complement C3. The hub chemokines CCL5 and CCR2 recruited macrophages [18, 19], which could produce CXCL1 [20], while CCL5, CCL19, and CCL21 recruited T cells [21–24]. This is consistent with the pathological observation that macrophages and T cells are the chief inflammatory infiltrates in DN [25–27]. Novel treatment targeting macrophages, inhibitors of CCL2 and CCR2 had been reported to successfully reduce proteinuria in DN [28, 29]. The increased mRNA expression of C3 in our study may suggest increased local production of the complement component. Plus, C3 ranked as one of the hub genes and highly interacted with proinflammatory factors in PPI, suggesting that C3 could aggravate DN inflammation by pathways besides complement activation. Previous study reported the increased transcriptome and protein levels of C3 in a part of diabetic patients with impaired renal function [10, 30]. And C3 positivity on renal histopathology correlated with severer kidney damage [30], whereas blockage of C3 signaling improved renal outcomes in various DN animal models [31]. Our results indicated that inflammatory infiltrates, particularly macrophages and T cells, and locally synthesized C3 could play key roles in the progression of DN.

One main pathological characteristic of DN is diffuse extracellular matrix accumulation. Both glomerular and tubular basement membrane thickening occur at an early stage [32, 33]. And in developed DN, glomeruli are often enlarged due to the increase of mesangial matrix, which further could result in nodular sclerosis [34, 35]. Meanwhile, tubulointerstitial fibrosis is common, and the interstitial volume is also increased with collagen and other matrix components [36]. In accordance with the pathological presentation, in our study, the GO annotation of upregulated DEGs in both glomeruli and tubulointerstitium demonstrated that extracellular matrix organization, extracellular space, and extracellular matrix structural constituent were predominantly enriched, suggesting the exceptionally active profibrotic process in DN. This was also evidenced by a more recent genome-wide association study in diabetic kidney disease, which highlighted biology involved in glomerular basement membrane collagen [37]. The nonresolving profibrotic process of DN has been well acknowledged as a pathogenetic mechanism leading to ESRD in DN [32, 38].

Moreover, GO analysis showed that both glomerular and tubulointerstitial DEGs were enriched for genes located in extracellular vesicles, including exosomes and blood microparticles. Extracellular vesicles are host cell-derived packages of information which mediate intercellular communication [39]. Accumulating evidence indicated that the levels of kidney-derived exosomes and blood microparticles elevated in diabetic patients [40–43]. Previous study reported that endothelial and podocyte extracellular vesicles had profibrotic effects on mesangial and tubular cells [44, 45], and tubular extracellular vesicles could induce inflammation [46]. It is also demonstrated that platelet microparticles in diabetic patients contributed to endothelial injury by extracellular communication [47]. And our analysis showed that increased DEGs in exosomes and blood microparticles mainly involved in leukocyte migration, complement activation, extracellular matrix organization, and platelet degranulation, which further supported the roles of extracellular vesicles mediated intercellular communication in the process of DN inflammation and fibrosis.

Injury of renal tubular cells is a prominent histopathological feature of DN and is regarded as an important contributor to impaired kidney function [48]. Exploring key pathways leading to tubular injury, in addition to exacerbated inflammation as discussed above, we found that the downregulated DEGs might have renoprotective effects as they positively correlated with eGFR. These decreased DEGs were significantly enriched in pathways associated with metabolism process and oxidation reduction process according to GO enrichment analysis. And the results of hub genes were similar, with function relating to metabolism process particularly lipid metabolism, i.e., EHHADH, HAO2, PECR, ACOX2, SLC27A2, and oxidation-reduction process, i.e., PECR, DHRS4 DAO, PIPOX. Downregulation of the lipid metabolism genes could result in tubule epithelial lipid accumulation, which is common in human DN and correlated with deterioration of renal function [49, 50]. Normally, tubular epithelial cells rely on fatty acids as energy source [48], while defective fatty acid utilization cause energy depletion. Tubular epithelial cells have high levels of baseline energy consumption and substantial mitochondria, and energy depletion would result in excess oxidative stress, subsequent injury, and even cell death [51, 52]. In our study, the PPI network of decreased DEGs also showed close interactions between lipid metabolism and oxidative stress. These results suggested that dysregulation of lipid metabolism and oxidative process could coordinately contribute to the tubulointerstitial impairment in DN.

Our study has several limitations. First, due to lack of detailed information of patients, it is difficult to control and evaluate the influence of demographic factors or the stage of the disease process on the analysis. Second, renal function impairment among different groups was unmatched. Third, the analysis was based on transcripts, which are not always the same as those on the protein level. Fourth, shared DEGs not enriched in enrichment analysis or selected as hub genes could also play an important role in the DN progression. However, as conventional treatment based on strict control of hypertension, hyperglycemia, and proteinuria fails to prevent DN patients from renal failure, novel therapeutic strategies are urgently needed. And this analysis suggests that strategies focus on impeding renal inflammation and fibrosis, correcting dysregulated tubular lipid metabolism and oxidative-reduction process from an integrative bioinformatics aspect.

## 5. Conclusions

In summary, our study disclosed the pathogenic molecules and pathways promoting the progression of DN independent of proteinuria. We identified a total of 566 glomerular and 581 tubulointerstitial shared DEGs and highlighted the importance of pathways associated with fibrosis, inflammation, lipid metabolism, and oxidative stress in DN. These genes and pathways could be potential targets for the treatment of DN.

## Data Availability

Data is available on request from the corresponding author of this article.

## Supplementary Materials

Supplementary Materials Supplementary File 1: Complete lists of DEGs (Fold change >1.5) in glomerular and tubulointerstitial compartments between DN versus healthy controls, MN or MCD. Supplementary File 2: GO annotation for upregulated DEGs in glomeruli and tubulointerstitial compartments, and downregulated DEGs in tubulointerstitium. Supplementary File 3: GO annotation of biological process for upregulated glomerular and tubulointerstitial DEGs enriched in extracellular vesicles.
